# Deep Submicron EGFET Based on Transistor Association Technique for Chemical Sensing

**DOI:** 10.3390/s19051063

**Published:** 2019-03-02

**Authors:** Salvatore A. Pullano, Nishat T. Tasneem, Ifana Mahbub, Samira Shamsir, Marta Greco, Syed K. Islam, Antonino S. Fiorillo

**Affiliations:** 1 Department of Health Sciences, University “Magna Græcia” of Catanzaro, 88100 Catanzaro, Italy; marta.greco@unicz.it (M.G.); nino@unicz.it (A.S.F.); 2 Department of Electrical Engineering, University of North Texas, Denton, TX 76203, USA; NishatTarannumTasneem@my.unt.edu (N.T.T.); Ifana.Mahbub@unt.edu (I.M.); 3 Department of Electrical Engineering and Computer Science, University of Missouri, Columbia, MO 65211, USA; sshamsir@mail.missouri.edu (S.S.); islams@missouri.edu (S.K.I.)

**Keywords:** EGFET, electronic interface, biosensors, low noise design, MOSFET, compact modeling, pH sensor

## Abstract

Extended-gate field-effect transistor (EGFET) is an electronic interface originally developed as a substitute for an ion-sensitive field-effect transistor (ISFET). Although the literature shows that commercial off-the-shelf components are widely used for biosensor fabrication, studies on electronic interfaces are still scarce (e.g., noise processes, scaling). Therefore, the incorporation of a custom EGFET can lead to biosensors with optimized performance. In this paper, the design and characterization of a transistor association (TA)-based EGFET was investigated. Prototypes were manufactured using a 130 nm standard complementary metal-oxide semiconductor (CMOS) process and compared with devices presented in recent literature. A DC equivalence with the counterpart involving a single equivalent transistor was observed. Experimental results showed a power consumption of 24.99 mW at 1.2 V supply voltage with a minimum die area of 0.685 × 1.2 mm^2^. The higher aspect ratio devices required a proportionally increased die area and power consumption. Conversely, the input-referred noise showed an opposite trend with a minimum of 176.4 nV_rms_ over the 0.1 to 10 Hz frequency band for a higher aspect ratio. EGFET as a pH sensor presented further validation of the design with an average voltage sensitivity of 50.3 mV/pH, a maximum current sensitivity of 15.71 mA^1/2^/pH, a linearity higher than 99.9%, and the possibility of operating at a lower noise level with a compact design and a low complexity.

## 1. Introduction

In the last 30 years, semiconductor-based biosensors have been amply investigated because of the numerous advantages introduced by innovative transducer materials and integrated circuit (IC) technology in terms of miniaturization and mass production [1]. Solid-state biosensors have found different applications in medicine, food, and environmental monitoring, especially with the most recent increasing concerns on food safety and health issues [2,3]. In this context, a field-effect transistor (FET) front-end electronic interface has been significantly investigated in a wide range of monolithic devices, such as an extended-gate field-effect transistor (EGFET) and an ion-sensitive field-effect transistor (ISFET). The main applications involve the detection of pH, urea, glucose, Ca^2+^, and DNA, as well as the fabrication of high frequency ultrasonic and piezoelectric sensors [4,5,6,7,8,9,10,11,12].

EGFET evolved from ISFET technology and was first introduced in 1983 by Van der Spiegel as its valid counterpart [13]. Although EGFET and ISFET have a similar design, the main difference is that for EGFET, the sensing membrane is far from the FET device, avoiding direct contact with the chemical environment. In the ISFET, the gate dielectric is in contact with an electrolyte, biased by a reference electrode acting as the gate, while in EGFET, the sensitive layer is characterized by low impedance (higher sensitivity) and the potential at the gate interface influences the drain current [14]. Thus, the activity of the target analyte results in an additional chemical contribution to the threshold voltage [15]. Since its introduction, EGFET has become an alternative to ISFET for improving performance in terms of threshold voltage drift, stability, responsiveness, and light/temperature sensitivity. In addition, the fabrication technology allows better device passivation, packaging, and lower mass-production cost [16,17].

One of the main features of monolithic EGFET is the design of a high aspect ratio device (higher transconductance and a lower noise), improving the performance for low-frequency applications [13,18]. However, most of the applications found in the literature use commercially available n–channel metal–oxide semiconductor field-effect transistors (MOSFETs) to implement EGFET biosensors [19,20,21,22]. Custom design of monolithic FET results in arduous sensor development in terms of effort, time, and cost with significant variability, also requiring biological modification directly on the channel region for improved performance [23]. CMOS technology is actually a consolidated technology with an easier single-chip and array integration, characterized by reduced variability, noise, and wiring complexity [23,24]. Moreover, since it does not require post-processing, multi-purpose CMOS technologies have been used to fabricate EGFETs [25,26].

Transistor association (TA) is a design technique investigated for analog circuits characterized by significant area savings, exploiting proper series/parallel connection of MOSFETs. The technique allows the design of low-voltage analog front-ends giving rise to higher DC gain, lower noise, and a higher cut-off frequency [27,28]. In this work, a monolithic EGFET design based on the TA technique is presented. High aspect ratio devices using standard deep submicron CMOS technology have been designed and fabricated by acting on the series/parallel association of transistors. Three different prototypes were fabricated and a comparison with the devices published in the current literature is presented. The TA method has been investigated as a valuable tool for the design of monolithic devices with reduced area and has a simple modeling compared to custom design. A further experimental validation as pH sensor of the design has been investigated. The paper is focused on the electrical performance of the proposed EGFET-based chemical sensor compared with its counterparts reported in the literature.

The paper is organized as follows: Section 2 discusses the EGFET design architecture, Section 3 presents the simulation and measurement results of the fabricated EGFET, while in Section 4 results are discussed. Finally, a concluding remark is included in Section 5.

## 2. Materials and Methods

### 2.1. EGFET Basic Principle 

**Figure 1:**
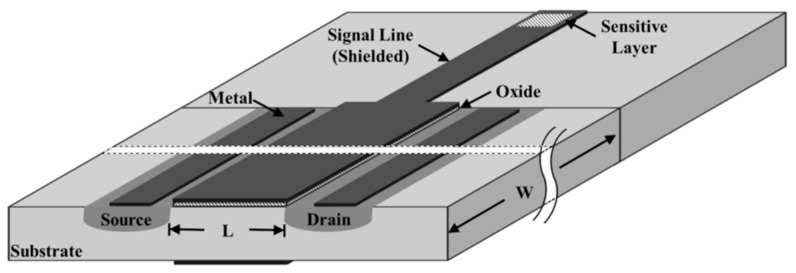
Schematic of a monolithic extended-gate field-effect transistor in which the sensitive interface is placed far from the metal–oxide semiconductor field-effect transistor (MOSFET).

The structure of the EGFET, as shown in Figure 1, is comprised of two parts: one is the chemically sensitive layer and the other one is the MOSFET. The sensing membrane is usually submerged in the buffer solution, while the FET device is kept at a certain distance from the solution to avoid contact with the chemical environment. The standard readout circuit of an EGFET sensor can be accomplished by a source follower.

The channel current of the MOSFET, *I_D_*, in the linear region can be written as follows:

(1) ID=μCoxWL[(VRef−Vth*)VDS−12VDS2]

where *μ* is the charge carrier mobility, *C_ox_* is the gate oxide capacitance per unit area, *W* and *L* are the channel width and length, respectively, and *V_Ref_* and *V_DS_* are the reference electrode and the drain-to-source voltages, respectively. V^*^_th_ represents the overall threshold voltage, which considers the activity of the target analyte, too [29]. A higher aspect ratio (*W/L*) results in a higher transconductance $g_{m}$ and reduced flicker noise. As far as the noise is concerned, an empirical model for the evaluation of the power spectral density (PSD) of the flicker noise current is expressed by the following relationship:

(2) $$S_{V_{G}}=\frac{q^{2}kT}{\gamma WLC_{ox}^{2}f}{\left[1+\alpha_{SC}\mu C_{ox}\left(V_{G}-V_{th}\right)\right]}^{2}N_{t}$$

where *f* is the working frequency, *k* is the Boltzmann constant, *T* is the temperature, *γ* is the attenuation coefficient of the electron wave function in the oxide, *α_sc_* is the Coulomb scattering parameter, and *N_t_* is the trap density [30,31]. Thermal noise plays an important role depending on the aspect ratio and the region of operation of the EGFET. Several models have been proposed for the modeling of the thermal noise. Tedja et al. proposed a model valid for “long-channel” devices, which highlights that an *n*–channel MOSFET, due to the larger body effect, has a higher noise level except for the strong inversion region where the effect becomes less significant with respect to a *p*–channel device [32]. The thermal noise of a MOSFET can be modeled as:

(3) $$S_{V_{G}}=4kTR_{equ}$$

where *R_equ_* is the gate-referred equivalent noise resistance. Noise reduction can thus be obtained by properly biasing the bulk/well and designing a symmetric layout. EGFET sensors do not significantly suffer from other external interferences due to the coupling from high-to-low impedance since it is physically located in close proximity to the reaction site [33]. As the final aim is the fabrication of a high aspect ratio device using standard CMOS technology, mainly by increasing the width with respect to the length, a reduction in the channel resistance is achieved. The use of the shorter channel MOSFET, however, involves a higher threshold voltage dependence [34].

### 2.2. Circuit Design

**Figure 2:**
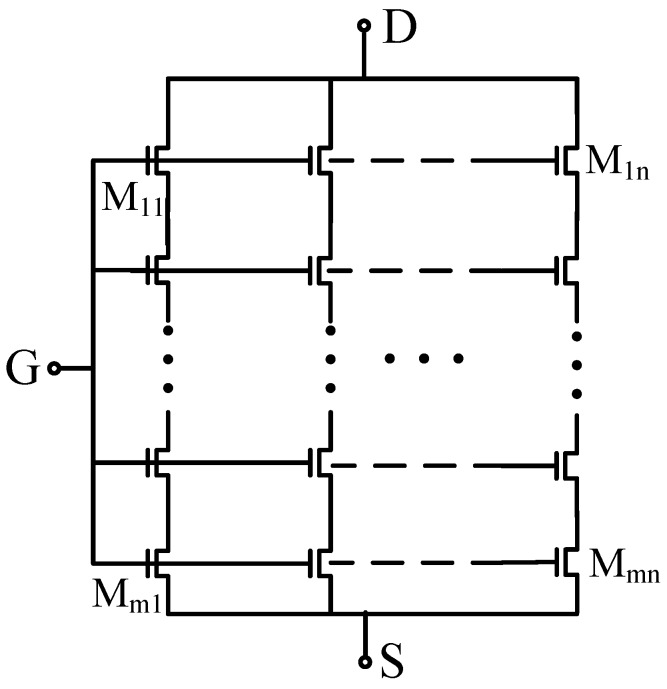
Schematic of the extended-gate field-effect transistor (EGFET) topology based on transistor association technique. Each element is characterized by a fixed *W/L* ratio.

The series/parallel association of transistors affects the performance of the equivalent device and has been intensively investigated for low-power analog circuits and power devices. Many industrial off-the-shelf MOSFET components are comprised of a large array of transistors in order to relax the overall constraints in terms of design, dimensions, time response, and power consumption [27,35]. A composite transistor-based EGFET is proposed in this work to achieve a high aspect ratio, as shown in Figure 2. In the first approximation, the composition of two identical transistors connected in series can be modeled in DC as a single transistor with half the aspect ratio, while in the case of a parallel combination, it gives rise to double the aspect ratio. Considering transistors with the same technological parameters (e.g., aspect ratio and short-channel effects), the overall equivalent width and length of the matrix of transistors can be written as:

(4) $${\left(\frac{W}{L}\right)}_{eq}=a\frac{W}{L}$$

where *a* = *n*/*m* is the ratio between the number of row elements *n*, divided by the number of column elements *m*, as can be seen from the architecture of the EGFET with transistor association technique.

Apart from the DC behavior, the array-based transistor will also impact the noise performance and the dynamic behavior with respect to the equivalent transistor [27]. As far as the thermal noise is concerned, the contribution is dependent on the gate-referred equivalent noise resistance and, thus, the (*W*/*L*)*_eq_*. In this case, the model results for TA and a single transistor are consistent and no significant variations are expected. On the other hand, flicker noise is influenced by the association of transistors. As reported in the literature, the flicker noise model shown in Equation 2 is consistent only for the parallel combination. In the work proposed by Arnaud et al., a simple model has been introduced considering the transistor association as follows [28]:

(5) $$S_{V_{G}}≅\frac{q^{2}N_{t}}{WLC_{ox}^{2}}\frac{1}{f}$$

Moreover, TA positively affects the dynamic behavior (e.g., cut-off frequency) [27]. The proposed analog front-end for EGFET sensors is designed as an *m* row by *n* column matrix of MOSFETs following the schematic as shown in Figure 2 in which *m* = 10 and *n* is equal to 475, 950 and 1900, respectively. Each single transistor is characterized by a fixed *W/L*, resulting in devices with an increasing array dimension.

### 2.3. pH Sensor

pH is a widely monitored parameter in biological and biochemical processes. The transduction mechanism of an electric double layer composed of an oxide-electrolyte interface is described by the site-binding model [26,36]. The pH of the solution influences the hydroxyl groups (or similarly the H^+^ ions) at the surface. The number of binding sites on the sensitive layer (which is a characteristic of the material) influence the potential of the electrolytic interface, as well as the potential of the sensing membrane. The potential between a reference electrode and the sensitive layer can thus be related to the pH of the solution [26].

The sensitivity of pH sensors is mostly affected by the buffer capacity of the sensitive layer (e.g., most common materials used are indium tin oxide (ITO), tin oxide (SnO_2_), titanium dioxide (TiO_2_), etc.), which leads to a maximum theoretical value known as Nernst limit of 59 mV/pH at 297 K, even though recent investigations have reported materials (e.g., palladium oxide (PdO)) that exceed this limit [37,38].

**Figure 3:**
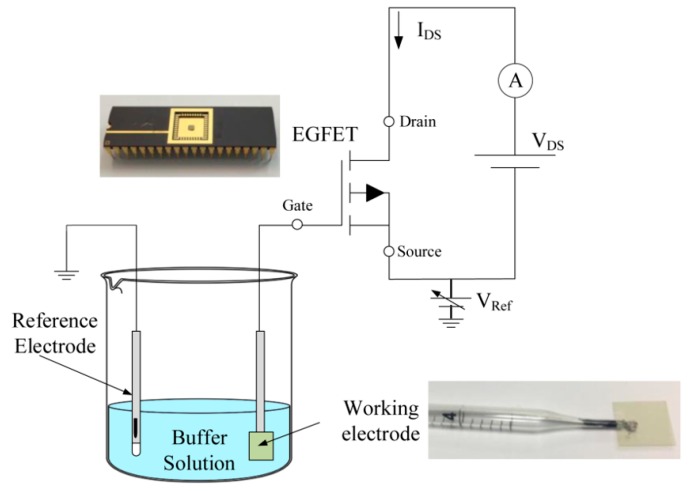
Schematic of the EGFET experimental setup.

Figure 3 shows the schematic diagram of an EGFET sensor setup for pH measurement. A buffer solution was used to characterize the EGFET in the range of pH from 2 to 12. Distilled deionized water, hydrochloric acid (HCl) 10 M, and sodium hydroxide (NaOH) 10 M were used to adjust the pH of the solution. The pH was monitored with a time-step of 5 seconds by using a commercial pH sensor (PH-BTA, Vernier Corp.), which is characterized by a response time of 1 s (90% of final reading) and an accuracy of ±0.2 pH. ITO-coated glass (Diamond Coatings, UK) was used as it is a commercial and inexpensive electrically conductive sensitive layer characterized by a dimension of 18 mm × 18 mm, a thickness of 1 mm, and a resistance of 15–30 Ω/sq. The ITO sample was bonded using an epoxy layer into a plastic pipette in which the conductive wire (i.e., coaxial cable) was connected. The plastic pipette created a homemade package to encapsulate the ITO layer and a conductive signal line while exposing a sensing area of approximately 2 cm^2^. Electric contact was made using a silver-loaded epoxy paste. The potentiometric cell configuration was referenced to a commercial Ag/AgCl electrode. The setup was composed of a programmable power supply (GWINSTEK GPD3303S) to set the *V_DS_* and *V_Ref_* and an electrometer (Keithley 6517B) for the evaluation of *I_D_* current.

## 3. Results

### 3.1. Simulation and Measurements on EGFET

**Figure 4:**
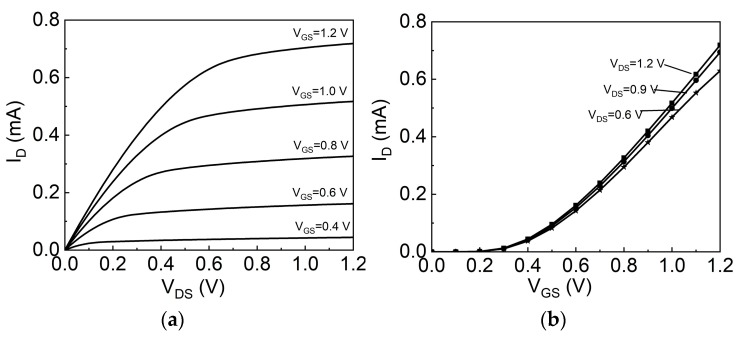
(**a**) Characteristics *I_D_*–*V_DS_* and (**b**) *I_D_*–*V_GS_* curves for a single transistor element with *W/L* = 4.21/1 µm/µm.

**Figure 5:**
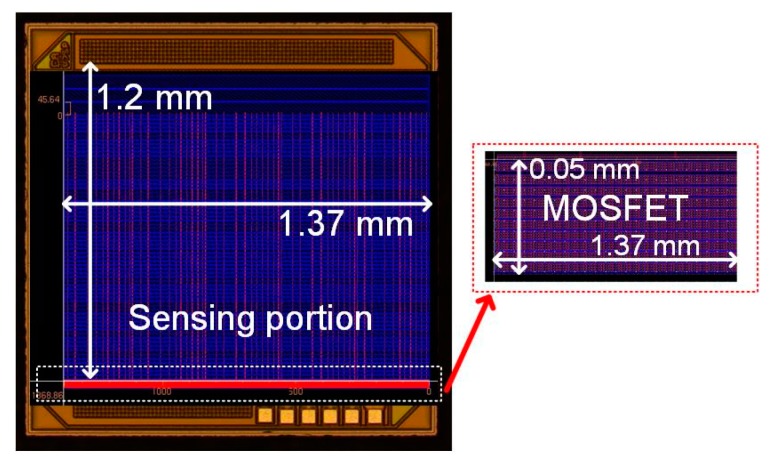
Representative chip microphotograph of one of the EGFET characterized by (*W*/*L*)*_eq_ = 95 W/L* µm/µm. The sensitive layer can be placed within the sensing portion area or externally in a separative EGFET (SEGFET) configuration [26].

Prototypes were fabricated in a standard 130-nm CMOS process in which each transistor unit was characterized by a width of 4.21 µm and length of 1 µm. The simulated output and the transfer characteristic curves for the single transistor element are shown in Figure 4, while in Figure 5, the chip microphotograph with the layout of one of the prototypes having a die area of 1.371 × 1.2 mm^2^ is shown.

**Figure 6:**
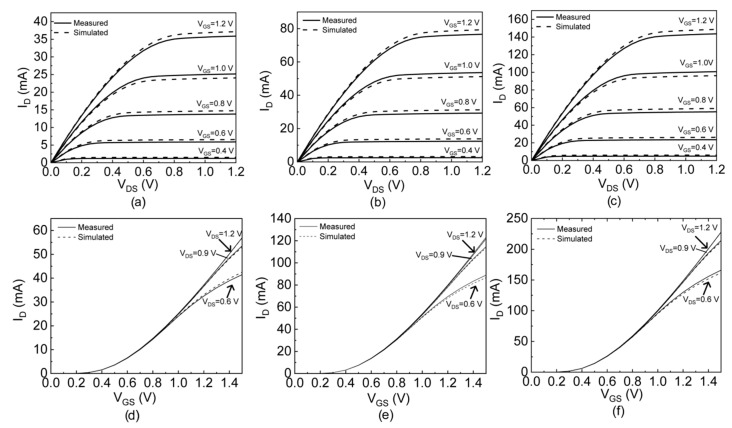
Simulated and experimental output and input characteristics for (**a**,**d**) (*W*/*L*)*_eq_* = 47.5 *W*/*L*, (**b**,**e**) (*W*/*L*)*_eq_* = 95 *W/L*, and (**c**,**f**) (*W*/*L*)*_eq_* = 190 *W/L*.

The simulated and the experimental DC output (see Figure 6a–c) and input (see Figure 6d–f) characteristics of each device are reported highlighting the agreement between the simulation and the experimental analysis. The linearity on the input characteristics (e.g., for the sensing performance of the EGFET) was analyzed on the *I_D_*–*V_GS_* curves for three *V_DS_* values (0.6, 0.9 and 1.2 V). The output characteristics were evaluated in the linear and saturation regions for the assessment of pH sensor sensitivity and linearity. The analysis on the input characteristics of Figure 6 evidenced the experimental threshold voltages of 256.2 mV, 260.9 mV, and 262.5 mV, respectively, for the three devices. These reported values are consistent with that of the single transistor (*V_th_* = 252.2 mV).

**Figure 7:**
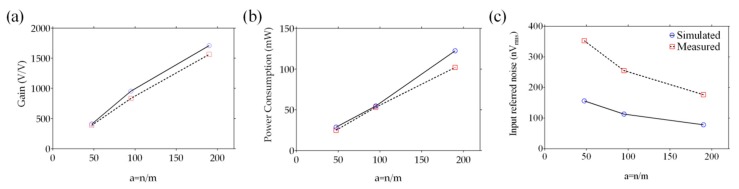
Simulated and experimental characteristics of the designed EGFETs: (**a**) voltage gain, (**b**) power consumption, and (**c**) input referred noise.

The three devices were also characterized in terms of the equivalent voltage gain *A_V_*, the static power consumption, and the overall input-referred noise. The prototypes were tested as voltage amplifiers. Voltage gain *A_V_* = $g_{m}R_{D}$ has been evaluated by using a load resistance *R_D_* = 10 kΩ with $g_{m}$ = *I_D_/V_GS_*. Figure 7a shows both the simulated and the measured voltage gains. Both the gate-to-source voltage *V_GS_* and drain-to-source voltage *V_DS_* were kept at 1.2 V. As expected from Figure 7a, the gain was the highest for the highest aspect ratio devices because of the increased $g_{m}$. The voltage gain of the devices work for a linear range of the *V_GS_* voltage from 0.4 V to 1.2 V for *V_DS_* voltage of 1.2 V. Since the static power consumption depends on the total drain current, it is higher for a higher aspect ratio, as evident from Figure 7b. The overall noise contribution was simulated over the frequency band of 0.1 Hz to 100 Hz as shown in Figure 7c. As the *W/L* ratio increased, the noise performance improved. The lower the noise of the electronic interface, the better was the performance of the EGFET sensor (e.g., limit of detection). A previous study has demonstrated how the EGFET is quite noise-free from external disturbances as the high-to-low impedance conversion takes place physically near the chemical reaction [33].

### 3.2. Characterization of EGFETs as pH Sensors

Experimental validation of the TA-based electronic interfaces was investigated through the evaluation of pH in a standard calibrated solution in the range 2 to 12. According to Equation (1), the *I_D_*–*V_Ref_* characteristics at a given *V_DS_* determine how the threshold voltage shift is related to the pH of the solution (higher pH is expected to result in a decreased threshold voltage). The pH voltage sensitivity of the ITO layer was evaluated from the *I_D_*–*V_Ref_* as:

(6) $$Voltage\;Sensitivity=\frac{\Delta V_{th}^{*}}{\Delta pH}$$

Similarly, the shift of output characteristics *I_D_–V_DS_* in saturation at a given *V_Ref_* were used for evaluating the pH current sensitivity. In this case, a downward shift of *I_D_* was expected as the pH of the solution increased. pH current sensitivity was evaluated as follows:

(7) $$Current\;Sensitivity=\frac{\sqrt{\Delta I_{D}}}{\Delta pH}$$

**Figure 8:**
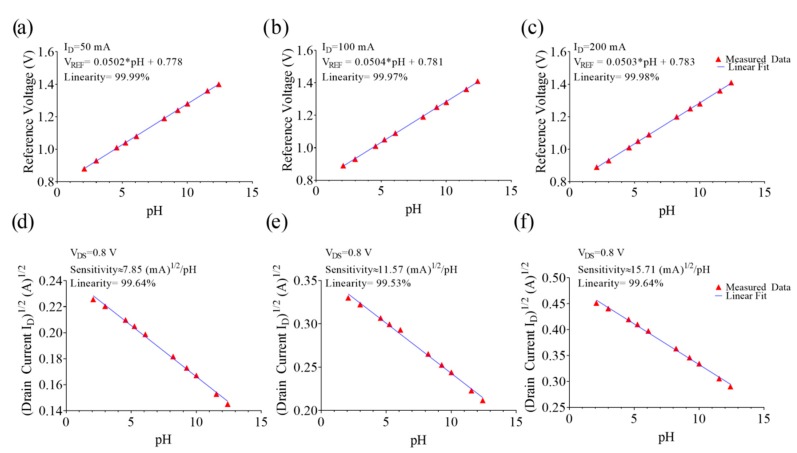
V_Ref_-pH response of the three EGFET-based prototypes evaluated in the linear region (*V_DS_* = 0.9 V) for (**a**) *I_D_* = 50 mA (a = 47.5), (**b**) 100 mA (a = 95), and (**c**) 200 mA (a = 190). I_D_-pH response of the three EGFET-based prototypes evaluated in the saturation region (*V_Ref_* = 0.9 V) for (**d**) a = 47.5, (**e**) a = 95, and (**c**) a = 190.

Figure 8 shows the results obtained from TA-EGFET devices in the linear region (see Figure 8a–c) using an increased drain current level, and at saturation (see Figure 8d–f) at a constant reference voltage.

While considering the linearity of the devices, the data were fitted in both the linear and saturation regions. In the linear region, the pH voltage sensitivity and linearity were evaluated from the *I_D_–V_Ref_* curves considering an *I_D_* of 50, 100, and 200 mA for the EGFETs with a = 47.5, a = 95, and a = 190, respectively. The experimental ranges were from 50.2 up to 50.4 mV/pH. In saturation, the current sensitivity and linearity were evaluated from the *I_D_*–*V_DS_* curves in response to pH variations at *V_Ref_* = 0.9 V. Here, the sensitivities according to Equation 7 were 7.85, 11.57, and 15.71 mA^1/2^/pH for the prototypes with a = 47.5, a = 95, and a = 190, respectively. In both cases, a linearity higher than 99.9% was observed. Reusability of the EGFET sensor relies on the sensitive layer, which can be easily disconnected, reactivated/exchanged, and reconnected. Two different sensitive layers were investigated on each electronic interface for the evaluation of EGFET reproducibility. The average voltage sensitivities of 50.2, 50.1, and 50.0 mV/pH, respectively, were obtained. Table 1 summarizes the characteristics of the proposed EGFET device in comparison to other commercial and non-commercial FET.

## 4. Discussion

The fabrication in standard CMOS technology allows for easier implementation by avoiding the custom design of a monolithic device, providing faster biosensor development with the flexibility of having different gate areas with power and noise optimization [44]. Recent evidence shows that despite a significant number of works being reported on the development of EGFET sensors, investigations focused on the electronic interfaces (e.g., noise performances, scaling effect, etc.) are still scarce. This is mostly because of the use of commercial electronic devices, the variety of interfacial materials, and the functionalization techniques, which move the focus onto the overall sensor development [26,45]. For this reason, this paper attempts to focus the emphasis on the electronic interface rather than on the detection principle of biological/chemical species.

TA showed output characteristics similar to an equivalent single MOSFET both in the linear region (low mismatch due to short-channel effects) and in saturation (low mismatch on output conductance). Both output and input characteristics showed consistent results with a slight shift (which are, however, within the limit of 5% variation). Deviations from simulated results could be mainly because of transistor mismatches and variations in the process technology (e.g., mismatches in the aspect ratio of the devices). A linear behavior was achieved for a *V_DS_* of 0.9 and 1.2 V, while for *V_DS_* = 0.6 V, the range was reduced for all three prototypes. 

Simulated and experimental performance metrics have been compared in terms of gain, power consumption, and input-referred noise. Devices with a higher *W/L* ratio were characterized by higher $g_{m}$ and, thus, a higher equivalent voltage gain. As far as the power consumption is concerned, the experimental results were slightly higher than the simulated ones for devices with an aspect ratio of 2000/10 (*a* = 47.5) and 8000/10 (*a* = 190). The higher the *W/L* ratio for the EGFET, the better was the performance in terms of gain and input-referred noise, compromising the performance in terms of power consumption. In addition, the higher *W/L* ratio device required a larger area, which could be a limitation when area optimization is being considered. The characterization showed a power consumption ranging from 25 to 102 mW with 1.2 V supply with a minimum die area of 0.685 × 1.2 mm^2^, which is comparable or better than most of the devices reported in the literature, as illustrated in Table 1. Conversely, the input-referred noise decreased as the *W/L* increased with levels ranging from 176.4 to 352.7 nV_rms_. However, the noise was referred only to the FET devices and does not consider the transduction interface. Table 1 highlights the fact that the noise level of the proposed electronic interface showed a lower noise level compared to the other works found in the literature, indicating lower electronic noise on the sensor performance. The proposed design also showed a power consumption that is comparable with other commercial and custom electronic interfaces.

The EGFET characterization was performed as a pH sensor using an ITO electrode connected to the gate of each prototype and immersed in a buffer solution with known pH values. Even though pH measurement is a routine work, recent literature shows how pH evaluation can be employed in advanced and novel fields of application, such as the monitoring of polymerase chain reaction (PCR) and in commercial sequencers [46]. Table 2 compares the proposed EGFET-based pH sensor with other representative pH sensors, particularly emphasizing the sensing performance. The proposed sensor achieved good linearity over the pH range of 2 to 12 and comparable characteristics with respect to the most recent literature.

According to a site-binding model, the surface charge was altered due to the dissociation of ITO and a change in the proton concentration of the buffer solution. A higher pH resulted in a decreased threshold voltage with a quite constant voltage sensitivity from 50.2 to 50.4 mV/pH and a current sensitivity of 7.85, 11.57, and 15.71 mA^1/2^/pH for the three prototypes, respectively. High linearity (>99.9%) in the pH range from 2 to 12 has been observed. However, in saturation, the sensor was inherently nonlinear, unless considering the square root of I_D_. Regarding the pH sensitivity and linearity, the obtained results are consistent with data reported in the literature [26].

## 5. Conclusions

In this paper, a technique based on transistor association has been investigated for the design and fabrication of an EGFET pH sensor. Transistor association technique has been investigated as a valuable tool for easier EGFET fabrication at a variable W/L in a 130-nm CMOS process. The proposed designs have achieved a minimum die area of 0.685 × 1.2 mm^2^ and a power consumption of 24.99 mW with 1.2 V supply voltage. Moreover, the proposed electronic interfaces achieved a minimum input-referred noise of 176.4 nV_rms_ over the frequency band of 0.1 Hz to 100 Hz, which is much lower than the other representative interfaces reported in the literature. A pH application has been proposed using a commercial ITO electrode in a standard solution. The sensor showed an average voltage sensitivity of 50.3 mV/pH and a maximum current sensitivity of 15.71 mA^1/2^/pH with linearity higher than 99.9%. The proposed work demonstrates the possibility of improving the performance of EGFET-based sensors over the existing electronic interface designs by investigating the important points highlighted by Van der Spiegel in its original concept of monolithic sensors. Even though the proposed application was validated for pH detection, other sensing modalities can also be developed by meeting the actual scientific and commercial requirements similar to most of the developed commercial and non-commercial EGFET-based biosensors.

## Figures and Tables

**Table 1:** Characteristics of the proposed extended-gate field-effect transistor (EGFET) devices compared to commercial and non–commercial devices.

	[6,39,40]	[41,42]	[23]	[43]	This Work #1	This Work #2	This Work #3
**W/L** **(µm/µm)**	PMOS: 360/10 NMOS: 170/10	9700/2	18/1	NR	2000/10	4000/10	8000/10
**Type**	CD4007UB	BS170	CMOS	CMOS	NMOS	NMOS	NMOS
**Process** **(µm)**	C	C	0.35	0.13	0.13	0.13	0.13
**Input referred noise** **(nVrms)**	NR	NR	1.58E6	4258 (@100 Hz)	252.7 (0.1 to 10 Hz)	255.1 (0.1 to 10 Hz)	176.4 (0.1 to 10 Hz)
**Power** **(mW)**	100	350	NR	3.77	24.99	53.23	102.0

C = Commercial. NR = Not Reported.

**Table 2:** Main characteristics of representative EGFET-based pH sensors.

Electrode	Range	Sensitivity (mV/pH)	Linearity (%)	Reference Electrode	FET Device	Reference
ITO/PET	2.1–12.1	45.9–52.3	98.3–99.6	Ag/AgCl	CD4007UB	[5]
InGaZnO	2–10	59.5	99.7	Ag/AgCl	CD4007	[16]
V_2_O_5_	2–10	58.1 ± 0.8	nr	nr	CD4007UB	[17]
ITO/PET	2–12	50.1 ± 1.7	98.5	Ag/AgCl	CD4007CN	[20]
SnO_2_	2–12	56–58	nr	SCE	CD4007UB or LF356N	[29]
PdO	2–12	62.87 ± 2	99.97	Ag/AgCl	CD4007UBE	[38]
ITO	2–12	58	nr	SCE	CD4007UB	[39]
TiO_2_	1.8–12	59.89	93.50	Ag/AgCl	NDP6060L	[47]
AZO	1–13	57.95	99.98	Ag/AgCl	CD4007UB	[48]
ITO/SiO_2_/Nb_2_O_5_	3–13	59.2	99.48	Ag/AgCl	IC4007	[49]
SnO_2_/ITO/PET	2–12	53.8–58.7	nr	Ag/AgCl	LT1167–I.A.	[50]
Glass	2–12	55	nr	nr	CD4007UB	[51]
CNT	3–13	50.9	99.78	nr	nr	[52]
ITO	2–12	50.2	99.9	Ag/AgCl	130nm CMOS	This work

PET, polyethylene terephthalate; AZO, aluminum-doped ZnO; CNT, carbon nanotube; I.A., instrumentation amplifier; nr, not reported.
